# Industrial Colonies for Mental Defectives

**Published:** 1912-07-13

**Authors:** 


### THE DEFECTIVE AND DEPENDENT.*

### X.—Industrial Colonies for Mental Defectives.

In the progress of training the feeble-minded the necessity for providing life-long care for a considerable percentage of this class has become increasingly apparent.

> "'Tis not enough to help the feeble up,
> But to support him after,"

is a maxim quite apposite in these cases. On a small scale, as we have seen, attempts have been made to supplement the work of training institutions by a modicum of permanent care. But in view of the extent and urgency of the need, it would seem that the best solution of the problem is by the organisation of colonies of an industrial character, where industries acquired in youth may be carried on with more or less profit under life-long supervision. Segregation from the ordinary population is essential in many cases to diminish the risk of transmission to another generation of heritable mental weakness; at the same time a colony affords by its size a varied sphere of activity for its inmates, which would save them from monotony while protecting them from harm. As distinguished from the old type of residential institution, a colony affords better opportunity for distinctive classification and for the amenities of family life, inasmuch as the inmates live in comparatively small homes, scattered usually over a considerable acreage of land. It may be interesting to trace the evolution of such a colony, and that recently established by the National Association for the Feeble-Minded may be taken as an example.

### The Princess Christian Farm Colony.

The National Association had during the first fifteen years of its existence established three residential homes for feeble-minded girls, and in 1897 the first boys' home was formed by renting farm premises in Essex, where about twenty youths above school age were trained to agricultural and horticultural pursuits under the care of a master and matron. The land attached being of small extent, it was found that the trained labour available was more than could properly be utilised at home. The experiment was consequently tried of sending some of the more capable boys out to work for farmers in the vicinity. This plan did not prove altogether satisfactory for lack of adequate supervision, and after ten years' experience on a small scale the Association resolved to launch out into a larger enterprise, and purchased 171 acres for £11,000 suitable for dairy farming, near Hildenborough, in Kent. Two of the buildings thereon have been adapted for homes for the male inmates, each accommodating about twenty-five; and at a later stage it was deemed advisable to transfer one of the girls' homes from London to the colony. New buildings have been erected for the latter at a cost of about £60 per head, of course at a judicious distance from those occupied by the males; and there are now two homes, each for twenty-five girls, under the charge of matrons.

### Its Administration.

Married couples manage the boys' homes, and, throughout, the ideal is that of family life rather than mere institutional régime. The male inmates are employed in the work of the garden and farm, and also in such handicrafts as carpentry and shoemaking; and the girls do all the laundry work for the community (over 170 persons), and are engaged in domestic avocations and needlework. There is a resident medical superintendent responsible to the committee for the general management, and the masters of the colony and matrons are responsible to him for the due discharge of their duties. Games and entertainments are not forgotten, and the community may be described as cheerful and well contented with their surroundings. The majority of the inmates are paid for by Boards of Guardians at rates varying from 7s. 6d. to 12s. per week. Current maintenance expenses are thus met, but it must be remembered that the colony was established by charitable contributions and loans. There is, however, no reason why Boards of Guardians should not themselves establish similar colonies, as has already been done by a combination of the Birmingham Unions.

### Monyhull Colony.

In 1906 the Birmingham, Aston, and King's Norton Boards of Guardians combined to provide a

* Previous articles appeared on Nov. 4, 18, Dec. 2, Jan. 6, Feb. 10, March 2, 23, May 4, June 15.

joint colony for the care of (1) sane epileptics, and (2) feeble-minded persons chargeable to any of the Unions co-operating. They purchased an estate at Monyhull consisting of 185 acres of freehold land, a large house, lodge, and two farms at a total cost of £20,600. This was opened as a colony in 1908, and then consisted of three houses for men and three for women, each containing thirty-six inmates, with laundry, kitchen block, workshops, and administrative headquarters at Monyhull Hall. The males work in the workshops and on the land; the women in the laundry, the sewing-rooms, and also out-of-doors in the summer picking fruits and vegetables. The administration is in the hands of a matron-superintendent, and there is a non-resident medical officer. It is in contemplation to enlarge the scope of the colony by providing a separate department for children to be dealt with under the Education Act of 1899 (Defective and Epileptic Children), in which such children will be received not only from the Unions but on contract from the special schools of Birmingham. Thus a comprehensive colony, both for training the young and the permanent care and employment of adults, will ultimately be formed. A colony on these lines has indeed been at work for over ten years on a charitable foundation under the auspices of the Lancashire and Cheshire Association for the Permanent Care of the Feeble-Minded, called

### The Sandlebridge Colony.

The distinctive feature of this colony is that all the inmates are admitted when young—as far as possible not more than twelve years of age—and after special education in well-equipped schools pass naturally at sixteen and upwards into the life and work of the colony. There were 232 residents at date of last report (135 males, 97 females), of whom sixty-eight ranged from sixteen to twenty-two years of age. The estate extends 100 acres, and includes a well-stocked farm and gardens with greenhouses, and a profit of nearly £600 was yielded by the produce of the latter in the year 1910-11. The boys are also employed in handicrafts, and the girls in laundry work and machine-knitting, and a weaving shed for them is contemplated. The gross annual cost per head is stated as under £28, and the revenue is derived from payments by education authorities and Boards of Guardians, and parents, supplemented by charitable contributions, which alone bore the initial expense of forming the establishment.

